# Reliability and Safety of Smartwatch Blood Pressure and Oxygen Saturation Measurements in Older Adults: Instrument Validation Study

**DOI:** 10.2196/81955

**Published:** 2026-05-22

**Authors:** Ricardo Madeira, Dulce Esteves, Henrique Neiva, Adriana Maia, Catarina Rondão, Nuno Pinto, Alessandro Vercelli, Maria Vaz Pato

**Affiliations:** 1 Department of Sports Sciences, University of Beira Interior, R do Bairro da Nossa Sra da Conceição 22, Covilhã, 6201-001, Portugal, 351 962076801; 2 RISE Health UBI, Faculty of Health Sciences, University of Beira Interior, Covilhã, Portugal; 3 Research Center in Sports Sciences, Health Sciences and Human Development (CIDESD), Covilhã, Portugal; 4 Department of Neuroscience Rita Levi Montalcini, Neuroscience Institute Cavalieri Ottolenghi, Turin, Italy

**Keywords:** wearable, blood pressure, hypertension, arterial oxygen saturation, technology

## Abstract

**Background:**

Hypertension is a significant risk factor for cardiovascular diseases and premature mortality, with its prevalence increasing due to population aging and lifestyle factors. Accurate measurement of blood pressure (BP) and arterial oxygen saturation is crucial for disease prevention and monitoring, and wearable devices have emerged as a promising alternative. However, their clinical reliability requires validation, particularly in older populations.

**Objective:**

The aim of this research was to evaluate and compare the measurement of BP and arterial oxygen saturation in older people using a smartwatch in comparison with reference devices.

**Methods:**

We recruited 50 participants aged between 50 and 89 years (mean 70.60, SD 12.03 y), including 34 female participants and 16 male participants. A total of 3 BP measurements were taken simultaneously using the smartwatch and an ambulatory BP monitoring device (reference device). Arterial oxygen saturation was measured simultaneously using the smartwatch and the oximeter. The paired-sample *t* test (2-tailed) was used to compare variables, and the intraclass correlation coefficient (ICC) was used to verify the correlation.

**Results:**

When averaged values were considered, no significant differences were observed between the Samsung Galaxy Watch 6 and the reference device for systolic BP (*P*=.31) or diastolic BP (*P*=.88), with good agreement for both parameters (systolic BP ICC=0.88; diastolic BP ICC=0.88). Arterial oxygen saturation showed no significant difference between devices (*P*=.10), with moderate agreement (ICC=0.68). Heart rate measurements also showed no significant differences between devices (*P*=.54), demonstrating good agreement.

**Conclusions:**

The Samsung Galaxy Watch 6 demonstrated acceptable agreement with reference devices for BP and arterial oxygen saturation measurements in older adults without decompensated clinical conditions, evaluated under controlled resting conditions. These findings indicate that the device provides reliable measurements within this specific population and context when measurements are obtained under standardized and physiologically stable conditions.

## Introduction

Elevated blood pressure (BP) stands as a pivotal factor correlated with heightened susceptibility to cardiovascular ailments, cerebrovascular events, and premature mortality [1-4]. In 2019, elevated BP was causatively linked to 10.8 million annual deaths globally [3]. The prevalence of hypertension is on an upward trajectory worldwide, attributed to increased average longevity, advancing age demographics, and modifiable lifestyle determinants [3,4]. Advancing age emerges as a predictor of hypertension incidence across sexes [4]. Current estimates indicate that 68% of individuals aged 60 years and older are afflicted with hypertension [5], precipitating an escalation in global morbidity and mortality rates [3]. By 2019, approximately 700 million individuals had attained the age of 65 years or older [6], with projections indicating a doubling of this figure to 1.5 billion by 2050 [6,7]. Concurrently, this demographic shift is poised to engender a surge in hypertension prevalence and associated morbidities, exerting a significant societal burden [6-8].

Accurate BP assessment is essential for cardiovascular risk prevention and management [3,4]. Failure to validate BP measurement devices may result in diagnostic errors, leading to inappropriate clinical decisions [4]. Although office-based measurements remain the clinical reference standard, current guidelines recommend ambulatory or home BP monitoring to improve diagnostic accuracy and reduce phenomena such as white-coat hypertension [4,9]. Given the multifactorial and dynamic nature of BP, approaches that allow repeated or continuous assessment may provide additional clinical value [4].

Arterial oxygen saturation measurement is also clinically relevant, particularly for the early detection of hypoxemic events in acute and chronic cardiopulmonary conditions, including obstructive sleep apnea and respiratory diseases [10]. Monitoring arterial oxygen saturation supports patient triage and informs further diagnostic evaluation, contributing to timely and appropriate clinical management [10].

Technological advancements significantly contribute to health care by offering novel avenues for self-management [11]. Assistive technologies [12] and monitoring tools [6,13,14] play major roles in promoting active aging [6], facilitating prolonged independence for older adults in their residences [12]. Furthermore, continuous, noninvasive real-time monitoring of cardiovascular parameters such as heart rate (HR), BP, and peripheral oxygen saturation (SpO_2_) holds paramount importance [15]. Such ongoing monitoring enables early detection and prevention of various cardiac issues [15]. The Internet of Things emerges as a promising avenue for health monitoring through wearable devices [6,9]. These devices, which have garnered substantial attention in recent years, offer monitoring capabilities across a spectrum of parameters, including HR, BP, body temperature, blood oxygen saturation, stress levels, sleep patterns, fall detection, and physical activity [6,9,14-17]. With user-friendly interfaces and relatively affordable costs, coupled with advanced analysis and artificial intelligence techniques, wearable devices present an appealing and valuable option for health data extraction [6,9,14-17]. Such technological solutions, particularly in BP assessment, play a crucial role in hypertension management, especially in older individuals given the high prevalence of hypertension in this demographic [5].

With the global older population and the incidence of cardiovascular diseases on the rise, research into assistive technologies for older adults is experiencing rapid expansion [9]. These technologies serve as invaluable resources for promoting active aging and providing health care support in conditions such as dementia, cardiovascular diseases, frailty, and other age-related ailments [9,18]. While wearables offer numerous benefits for older adults and individuals susceptible to conditions like cardiovascular diseases, hypertension, diabetes, cardiac arrhythmia, hyperlipidemia, and sleep apnea, ensuring their reliability and safety for clinical use, particularly among this demographic, is paramount [19]. Therefore, both the World Health Organization (WHO) and research bodies advocate for the validation of such technologies using gold standards [19].

Therefore, this study aims to evaluate and compare BP measurements in older adults obtained using the Samsung Galaxy Watch 6 with those obtained using a clinically validated reference BP device (Tonoport; GE Healthcare) under controlled conditions. The objective is to assess the agreement and measurement reliability of the Samsung Galaxy Watch 6 for BP assessment. We intend to verify the reliability of the Samsung Galaxy Watch 6 as sufficiently accurate to be used as a BP measurement device. We also aim to evaluate the Samsung Galaxy Watch 6’s ability to measure arterial oxygen saturation.

## Methods

### Overview

This is a quantitative, descriptive, cross-sectional study aimed at verifying the reliability of wearable sensors (Samsung Galaxy Watch 6) for measuring BP and SpO_2_ in participants aged 50 years and older.

Individuals aged 50 to 89 years were enrolled, comprising Caucasian individuals of Portuguese nationality from the Beira Interior region. Participants were recruited from the general community through local public announcements (eg, community notice boards and local outreach) and by direct invitation. No recruitment was conducted through medical care facilities, and participants were not employees of specific companies or institutions. Exclusion criteria included the inability to calibrate the Samsung Galaxy Watch 6 and the presence of wrist tattoos. Manufacturer specifications deemed systolic values greater than 169 and diastolic values less than 50 unacceptable for smartwatch calibration. Given the potential for various illnesses and medication use in this age group, these factors were not considered grounds for exclusion. BP measurements were conducted between January 2, 2024, and February 1, 2024.

### Protocol Design

To validate the reliability of the Samsung Galaxy Watch 6 (SM-R930) in terms of BP, we used a clinically validated reference BP device (TONOPORT V D-10829) under controlled conditions. According to North American [20,21], European [22,23], Japanese [24], and Chinese [25] clinical guidelines, reference-grade BP measurement devices are considered the standard for accurate BP assessment [26]. The TONOPORT V has previously demonstrated good accuracy and reliability for systolic and diastolic BP measurements when compared with mercury sphygmomanometry, fulfilling all phases of the European Society of Hypertension International Validation Protocol [27]. However, in this study, the device was used exclusively to perform stationary, office-based BP measurements under controlled conditions.

### Calibration

For calibration purposes, we adhered to the manufacturer’s guidelines. Participants were instructed to abstain from alcohol, caffeine, smoking, and exercise for 30 minutes prior to calibration, ensuring dry skin [9]. Calibration occurred in a tranquil setting, with participants seated comfortably, backs supported, arms resting on a table, legs uncrossed, and feet flat on the floor. Participants rested in this position for 5 minutes before commencement. During calibration, participants were instructed to remain still and breathe normally. Using the Samsung Health Monitor application, on-screen instructions guided the calibration process. Each participant underwent an individual calibration protocol consisting of 3 consecutive BP measurements obtained using a clinically validated reference BP device (TONOPORT V D-10829). Calibration was exclusively required for the BP variable, as per the manufacturer’s standards and instructions.

### Installation of Measuring Devices

The cuff was installed in accordance with the device’s standards. Thus, the reference BP device (TONOPORT V D-10829) cuff was placed on the patient’s nondominant arm, at a height of about 2.5 cm above the elbow. The cuff would only be placed on the other arm if there were any limitations. The cuff was to fit snugly on the patient’s arm, but not too tightly [28].

Installation of the Samsung Galaxy Watch 6 followed the manufacturer’s instructions. In this way, the Samsung Galaxy Watch 6 was worn on the wrist contralateral to the one used for the reference BP device. The Galaxy Watch strap was appropriately fitted to the participant’s wrist without excessive tightness. Due to the placement of the reference BP device, it was decided to place the watch on the opposite arm to ensure that measurements did not conflict.

The pulse oximeter was placed on the patient’s right index finger, which was on the same wrist where the smartwatch was worn. To ensure an accurate reading, it was important to place the oximeter snugly against the patient’s skin. This meant that the sensor should be firmly attached to the skin, and there should be no gaps between the sensor and the skin. Once the oximeter was properly adjusted, we waited at least 30 seconds before taking the oxygen saturation reading. This allowed the sensor to acclimate to the patient’s temperature and blood flow. To ensure that painted fingernails would not cause erroneous readings, we placed the probe oriented laterally so that the sensor transmitted the light on the side of the finger [29].

### Measurements

The participant was subjected to a 5-minute rest (remaining seated). After resting for 5 minutes, we calibrated the Samsung Galaxy Watch 6 in relation to BP. After calibration, 3 BP measurements were taken with a 5-minute interval between each measurement. The reference BP device (TONOPORT V D-10829, Germany) and the Samsung Galaxy Watch 6 (SM-R930) were used to measure BP simultaneously.

For analytical purposes, BP data were evaluated using 2 complementary approaches. First, each measurement session was analyzed separately to assess within-session variability and agreement between the smartwatch and the reference device at each time point, without averaging across sessions. In addition to the analysis of each individual measurement session, particular emphasis was placed on the overall analysis calculated as the mean of the 3 consecutive measurements. This approach was adopted to reduce the influence of random variability and isolated measurement errors, which are common in single BP readings [22,23]. The use of averaged values is consistent with standard clinical practice, where digital BP devices typically rely on multiple consecutive measurements to improve accuracy and support clinical decision-making [22,23].

SpO_2_ was measured after the BP measurements. One measurement was taken simultaneously with the oximeter (Proficare PO 3104) and the Samsung Galaxy Watch 6. All BP and SpO₂ measurements were performed with participants in a seated position, resting comfortably in a chair with back support. Participants were instructed to keep both feet flat on the floor, legs uncrossed, and arms supported at heart level. A resting period was observed prior to measurements to ensure hemodynamic stabilization. The same body position was maintained throughout the measurement protocol.

### Statistics

Microsoft Office Excel 2013 and the statistical analysis software IBM SPSS version 28.0 were used for data analysis. The calculation of means, SDs, differences, and 95% CIs was based on standard statistical methods. To verify the normality of the data, the Kolmogorov-Smirnov test (n>30) was used. The paired-sample *t* test (2-tailed) was used to compare the variables. The degree of agreement between measurements was evaluated using the intraclass correlation coefficient (ICC), calculated with a 2-way mixed-effects model with absolute agreement and single measures. ICC values were interpreted as indicating low reliability when below 0.5, moderate reliability between 0.5 and 0.75, good reliability between 0.75 and 0.9, and excellent reliability when greater than 0.90. The significance level for rejecting the null hypothesis was set at an α level of .05. Using Bland-Altman graphs, the difference was calculated as the reference BP device minus the smartwatch measurements. All assessments obtained from the reference BP device and the smartwatch were used to construct the Bland-Altman graphs. The same procedure was also applied to SpO₂ measurements. An a priori sample size evaluation indicated that a sample of approximately 50 participants would provide 80% statistical power to detect small-to-moderate standardized differences between measurement methods (Cohen *d*=0.4), assuming a 2-sided significant α level of .05.

### Ethical Considerations

All procedures followed the guidelines of the Declaration of Helsinki and were approved by the research ethics committee of the University of Beira Interior (approval CE-UBI-Pj-2023‐064-ID1994). All participants signed a declaration of participation. Prior to participation, all individuals received detailed oral and written information regarding the study objectives, procedures, potential risks, and benefits, and they provided written informed consent. Participation was voluntary, and participants were assured of confidentiality and the right to withdraw from the study at any time without any consequences.

## Results

This study included 50 participants aged between 50 and 89 years (mean 70.60, SD 12.03 y), of whom 34 were female participants and 16 were male participants. Of the 50 participants, 12 were aged between 50 and 59 years (mean 54.67, SD 2.50 y), 12 between 60 and 69 years (mean 64.25, SD 2.60 y), 11 between 70 and 79 years (mean 76.09, SD 2.26 y), and 15 between 80‐ and 89 years (mean 84.40, SD 3.60 y). In the male group, the average age was 69.50 (SD 11.76) years, and in the female group, it was 71.12 (SD 12.30) years. The average BMI of the 50 participants was 26.15 (SD 3.93) kg/m^2^, with males (27.66, SD 2.43 kg/m^2^) having a higher BMI than females (25.44, SD 4.31 kg/m^2^). This characterization of the population is shown in Table 1.

**Table 1.** Population characterization.

Variables	N	Age (y), mean (SD)	BMI (kg/m^2^), mean (SD)
Age (y)			
50‐59	12	54.67 (2.50)	26.16 (4.86)
60‐69	12	64.25 (2.60)	25.03 (3.32)
70‐79	11	76.09 (2.26)	26.38 (3.73)
80‐89	15	84.40 (3.60)	26.87 (3.88)
50‐89	50	70.60 (12.03)	26.15 (3.93)
Sex			
Male	16	69.50 (11.76)	27.66 (2.43)
Female	34	71.12 (12.30)	25.44 (4.31)

As shown in Table 2, paired-samples *t* tests were performed to compare measurements obtained with the Samsung Galaxy Watch 6 and the reference device (Tonoport) across 3 consecutive measurement sessions. In addition to the analysis of each individual session, particular emphasis was placed on the overall analysis, calculated as the mean of the 3 measurements. This approach was adopted to minimize the influence of isolated measurement variability and is consistent with procedures commonly applied in clinical practice, where digital devices typically rely on averaged values from repeated measurements to support clinical decision-making.

**Table 2.** Data on trend measurements.

Trend measurement	Mean (SD)	SE (95% CI)	Median (IQR)	ICC^a^ (95% CI)	*P* value	ES^b^
SBP^c^						
1st measurement—Tonoport	139.26 (16.57)	2.34 (134.55-143.97)	139.00 (129.00-150.00)	0.87 (0.78‐0.92)	.006	0.403
1st measurement—Samsung Galaxy Watch 6	136.18 (14.67)	2.08 (132.01-140.35)	137.50 (126.00-145.00)	0.87 (0.78‐0.92)	.006	0.403
2nd measurement—Tonoport	134.38 (16.57)	2.34 (129.67-139.09)	136.00 (126.00-145.00)	0.90 (0.82‐0.94)	.78	–0.039
2nd measurement—Samsung Galaxy Watch 6	134.68 (16.61)	2.35 (129.96-139.40)	135.50 (124.00-145.00)	0.90 (0.82‐0.94)	.78	−0.039
3rd measurement—Tonoport	133.14 (17.08)	2.42 (128.29-137.99)	133.00 (125.25-146.00)	0.88 (0.79‐0.93)	.49	−0.097
3rd measurement—Samsung Galaxy Watch 6	133.94 (15.60)	2.21 (129.94-138.37)	134.00 (124.25-144.75)	0.88 (0.79‐0.93)	.49	−0.097
Tonoport—overall analysis	135.59 (16.84)	2.34 (134.55-143.97)	139.00 (126.25-147.00)	0.88 (0.84‐0.91)	.31	0.083
Samsung—overall analysis	134.93 (15.57)	2.08 (132.01-140.35)	137.50 (125.00-145.00)	0.88 (0.84‐0.91)	.31	0.083
DBP^d^						
1st measurement—Tonoport	77.56 (11.45)	1.62 (74.31-80.81)	79.00 (69.50-85.75)	0.82 (0.70‐0.89)	<.001	0.592
1st measurement—Samsung	74.16 (10.10)	1.43 (71.29-77.03)	74.00 (67.00-80.50)	0.82 (0.70‐0.89)	<.001	0.592
2nd measurement—Tonoport	71.54 (11.82)	1.67 (68.18-74.90)	72.50 (64.25-80.00)	0.90 (0.83‐0.94)	.02	−0.343
2nd measurement—Samsung	73.24 (11.03)	1.56 (70.11-76.37)	75.00 (65.50-79.75)	0.90 (0.83‐0.94)	.02	−0.343
3rd measurement—Tonoport	71.94 (11.01)	1.56 (68.81-75.07)	71.00 (65.00-79.00)	0.91 (0.85‐0.95	.02	−0.334
3rd measurement—Samsung	73.44 (11.08)	1.57 (70.29-76.59)	74.50 (65.00-80.00)	0.91 (0.85‐0.95)	.02	−0.334
Tonoport—overall analysis	73.68 (11.68)	1.61 (74.31-80.81)	74.00 (65.25-81.00)	0.88 (0.83‐0.91)	.88	0.012
Samsung—overall analysis	73.61 (10.68)	1.43 (71.29-77.03)	74.00 (65.50-80.00)	0.88 (0.83‐0.91)	.88	0.012
HR^e^ ABPM						
1st measurement—Tonoport	72.28 (11.97)	1.69 (68.88-75.68)	70.50 (63.00-80.00)	0.76 (0.61‐0.86)	.97	−0.005
1st measurement—Samsung	72.32 (9.39)	1.32 (69.65-74.99)	71.00 (66.00-79.50)	0.76 (0.61‐0.86)	.97	−0.005
2nd measurement—Tonoport	71.30 (10.16)	1.44 (68.41-74.19)	69.50 (62.00-80.00)	0.87 (0.79‐0.93)	.78	0.040
2nd measurement—Samsung	71.10 (9.69)	1.37 (68.35-73.85)	69.50 (65.00-78.75)	0.87 (0.79‐0.93)	.78	0.040
3rd measurement—Tonoport	70.14 (10.96)	1.55 (67.03-73.25)	68.50 (62.00-80.00)	0.67 (0.79‐0.93)	.30	−0.147
3rd measurement—Samsung	71.38 (9.78)	1.38 (68.60-74.16)	69.00 (64.25-79.00)	0.67 (0.79‐0.93)	.30	−0.147
Tonoport—overall analysis	71.24 (11.02)	0.90 (68.88-75.68)	70.50 (62.00-80.00)	0.76 (0.69‐0.82	.54	−0.050
Samsung—overall analysis	71.60 (9.58)	0.78 (69.65-74.99)	71.00 (65.00-79.00)	0.76 (0.69‐0.82)	.54	−0.050
Arterial oxygen saturation						
Pulse oximeter	96.66 (1.84)	0.26 (96.14-97.18)	97.00 (95.00-98.00)	0.68 (0.49‐0.80)	.10	0.239
Samsung	96.28 (2.10)	0.30 (95.68-96.88)	96.50 (95.00-98.00)	0.68 (0.49‐0.80)	.10	0.239
HR—arterial oxygen saturation						
Pulse oximeter	72.40 (11.04)	1.56 (69.26-75.54)	72.00 (65.00-80.00)	0.89 (0.81‐0.93)	.77	−0.041
Samsung	72.62 (11.14)	1.58 (69.46-75.78)	71.50 (65.25-80.00)	0.89 (0.81‐0.93)	.77	−0.041

a ICC: intraclass correlation coefficient.

b ES: effect size.

c SBP: systolic blood pressure.

d DBP: diastolic blood pressure.

e HR: heart rate.

f ABPM: ambulatory blood pressure monitoring.

It should be noted that paired-sample *t* tests were used as supplementary descriptive analyses and were not intended to assess agreement between measurement methods. The absence or presence of statistically significant differences in mean values does not, by itself, indicate agreement or disagreement between devices. Therefore, the interpretation of agreement in this study is primarily based on Bland-Altman analysis, limits of agreement, and absolute measurement differences expressed in clinically meaningful units.

For systolic blood pressure (SBP), a statistically significant difference between devices was observed only during the first measurement session (*P*=.006; effect size [ES]=0.403). No significant differences were identified in the second (*P*=.78; ES=−0.039) or third sessions (*P*=.49; ES=−0.097). When the overall mean values were considered, no statistically significant difference was found between the Samsung Galaxy Watch 6 and the reference device (*P*=.31; ES=0.083). Agreement between devices was consistently good, both across individual measurements (ICC ranging from 0.87 to 0.90) and in the overall analysis (ICC=0.88).

Regarding diastolic blood pressure (DBP), statistically significant differences were observed between devices in all 3 individual measurement sessions (first: *P*<.001, ES=0.592; second: *P*=.02, ES = −0.343; third: *P*=.02, ES=−0.334). However, analysis of the overall mean DBP values revealed no statistically significant difference between devices (*P*=.88; ES=0.012). Agreement between devices was classified as good in the first 2 sessions (ICC=0.82 and 0.90), excellent in the third session (ICC=0.91), and remained good when considering the overall analysis (ICC=0.88).

For HR measured during BP monitoring, no statistically significant differences were found between devices in any of the 3 sessions (*P*=.97, ES = −0.005; *P*=.78, ES=0.040; and *P*=.30, ES=−0.147). Similarly, no significant difference was identified in the overall analysis (*P*=.54; ES=−0.050). The intraclass correlation coefficients indicated good agreement in the first 2 sessions (ICC=0.76 and 0.87), moderate agreement in the third session (ICC=0.67), and good agreement for the overall mean values (ICC=0.76).

With respect to SpO₂, no statistically significant difference was observed between the Samsung Galaxy Watch 6 and the reference pulse oximeter (*P*=.10; ES=0.239). Agreement between devices was classified as moderate (ICC=0.68). Likewise, HR measured during SpO₂ assessment did not differ significantly between devices (*P*=.77; ES=−0.041), demonstrating good agreement (ICC=0.89).

Overall, although statistically significant differences were identified in some individual measurements, particularly for DBP, the analysis based on averaged values across repeated measurements, which reflects standard clinical measurement procedures using digital devices, demonstrated no significant differences and good agreement between the Samsung Galaxy Watch 6 and the reference instruments. Accordingly, greater emphasis was placed on the evaluation of systematic bias, limits of agreement, and the distribution of absolute differences, as these metrics provide a more appropriate assessment of agreement and clinical relevance in method-comparison studies than hypothesis testing alone.

**Figure 1.**
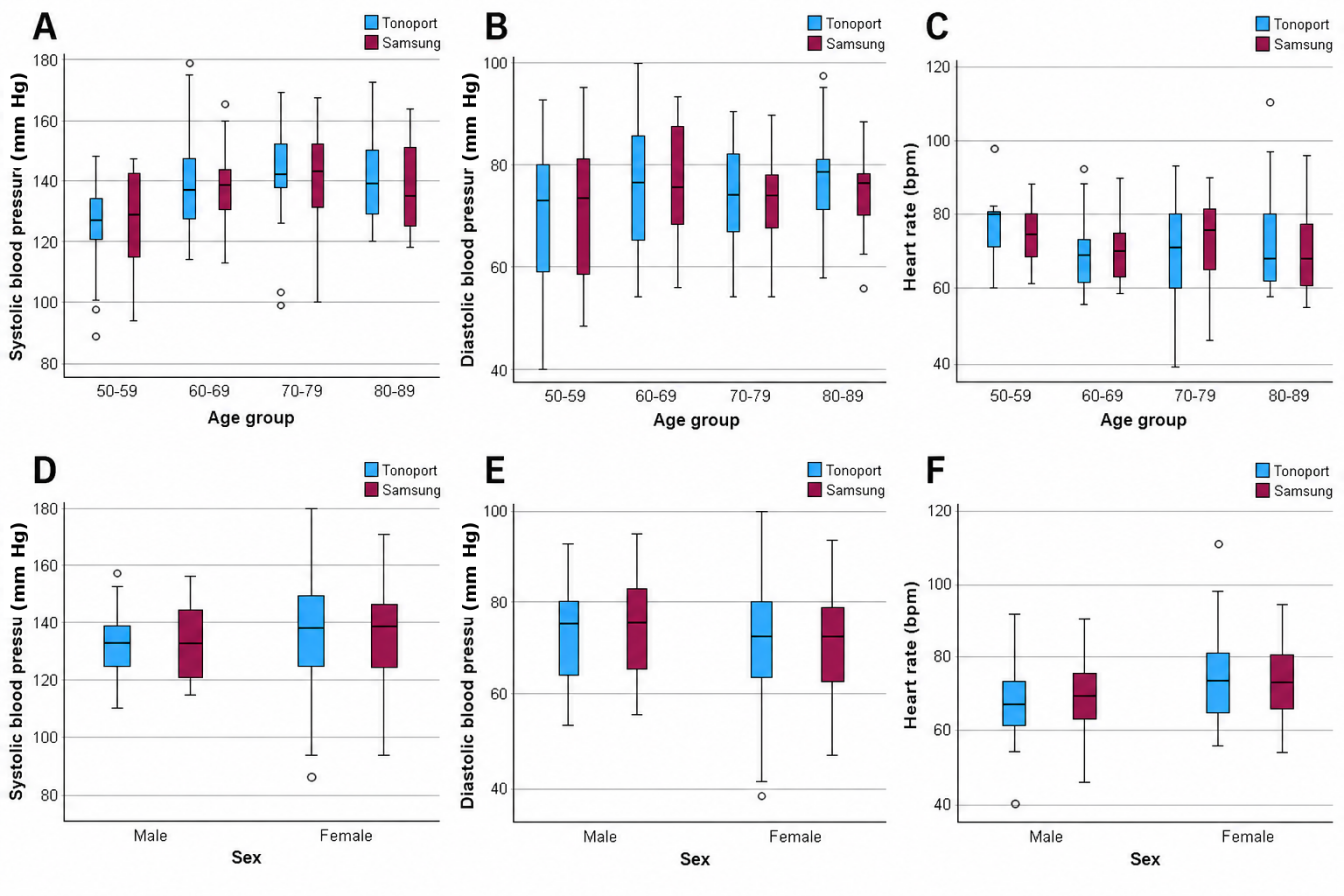
Boxplot of blood pressure measurements.

**Figure 2.**
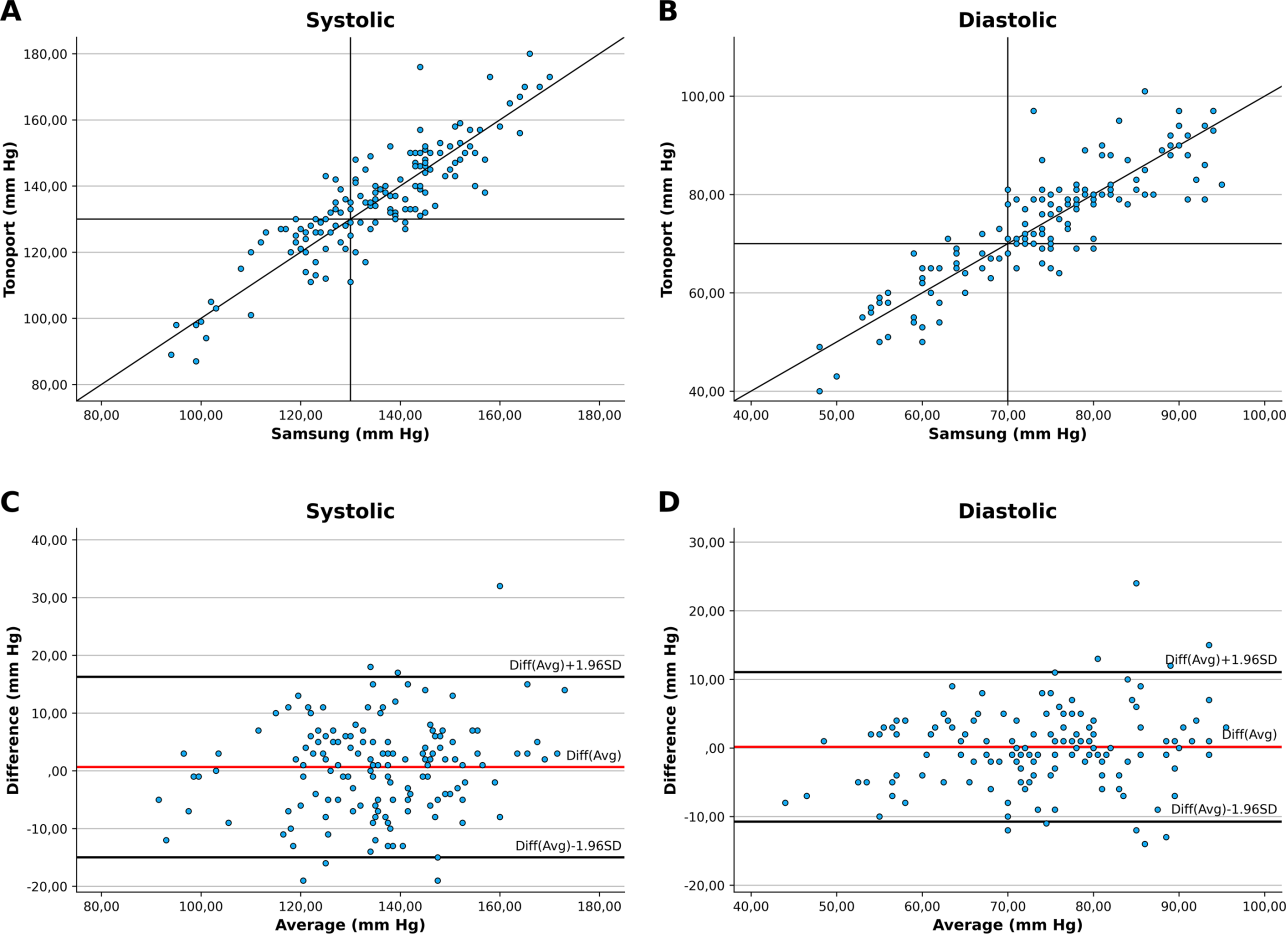
Scatterplots of blood pressure measurements.

According to Figure 1, there appear to be no differences between age groups or sexes. The boxplots (Figure 2) show that the variability is similar, with the first, second, and third quartiles, as well as the maximum and minimum, being identical for the 2 BP assessment methods.

Visual inspection of the scatterplots (Figure 2) revealed a clear positive linear relationship between BP values measured by the Samsung Galaxy Watch 6 and the reference BP device for both SBP and DBP. Most data points were distributed close to the line of identity, indicating that the smartwatch followed the same measurement trend as the reference method across the range of observed values.

Bland-Altman analysis demonstrated a small mean bias between the smartwatch and reference BP device measurements for both SBP and DBP, indicating no relevant systematic overestimation or underestimation by the smartwatch. Nevertheless, the limits of agreement were relatively wide, especially for SBP, reflecting considerable interindividual variability. For DBP, the limits of agreement were narrower, suggesting better agreement between methods compared with systolic measurements.

Overall, while the smartwatch demonstrated good agreement with the reference device at the group level, the Bland-Altman plots indicate that individual measurements may differ substantially, particularly at higher BP values.

The absolute differences between the reference BP device and Samsung are shown in Table 3. Table 3 indicates that most of the values between the 2 methods are less than 5 mm Hg. For SBP, 76% of the values are lower than 5 mm Hg, and for DBP, 88.7% of the values are below this threshold.

**Table 3.** Percentage of absolute blood pressure differences between the reference and the Samsung Galaxy Watch 6 within 5, 10, 15, and >15 mm Hg.

Blood pressure	≤5 mm Hg (%)	≤10 mm Hg (%)	≤15 mm Hg (%)	>15 mm Hg (%)
Systolic blood pressure	76	13.3	8.7	2
Diastolic blood pressure	88.7	8	2.7	0.6

In Tables4  5, BP was divided by 10 mm Hg according to the various BP parameters. In these tables, we can see that there is no difference between the various parameters. The biggest difference between the reference BP device and Samsung in SBP appears in values between 140 and 149 mm Hg, corresponding to a difference of 4.67%. In DBP, the biggest difference is between 70 and 79 mm Hg, corresponding to a difference of 9.33%. Using the Bland-Altman graphs, the difference was calculated using the reference BP device minus the smartwatch measurements. All the assessments made by the reference BP device and the smartwatch were used for constructing the Bland-Altman graphs.

**Table 4.** Percentage of absolute differences in systolic blood pressure between the reference and the Samsung Galaxy Watch 6.

Systolic	80-89 mm Hg	90-99 mm Hg	100-109 mm Hg	110-119 mm Hg	120-129 mm Hg	130-139 mm Hg	140-149 mm Hg	150-159 mm Hg	160-169 mm Hg	170-180 mm Hg
Tonoport (%)	1.33	2.67	2	5.33	22.67	26.67	19.33	14.67	1.33	4
Samsung (%)	0	2.67	3.33	8	21.33	23.33	24	12	4.67	0.67
Difference (%)	1.33	0	1.33	2.67	1.33	3.33	4.67	2.67	3.33	3.33

**Table 5.** Percentage of absolute differences in diastolic blood pressure between the reference and the Samsung Galaxy Watch 6.

Diastolic	40‐49 mm Hg	50‐59 mm Hg	60‐69 mm Hg	70‐79 mm Hg	80‐89 mm Hg	90‐99 mm Hg	100‐110 mm Hg
Tonoport (%)	2	10	22	33.33	24	8	0.67
Samsung (%)	1.33	10	18.67	42.67	18.67	8.67	0
Difference (%)	0.67	0	3.33	9.33	5.33	0.67	0.67

Considering the correlation between the SpO_2_ measured by the Samsung Galaxy Watch 6 and the oximeter in the SpO_2_ variable, the correlation is moderate (ICC=0.68). As for the correlation between HR measured by the oximeter and the watch, the correlation is classified as good (ICC=0.89).

**Figure 3.**
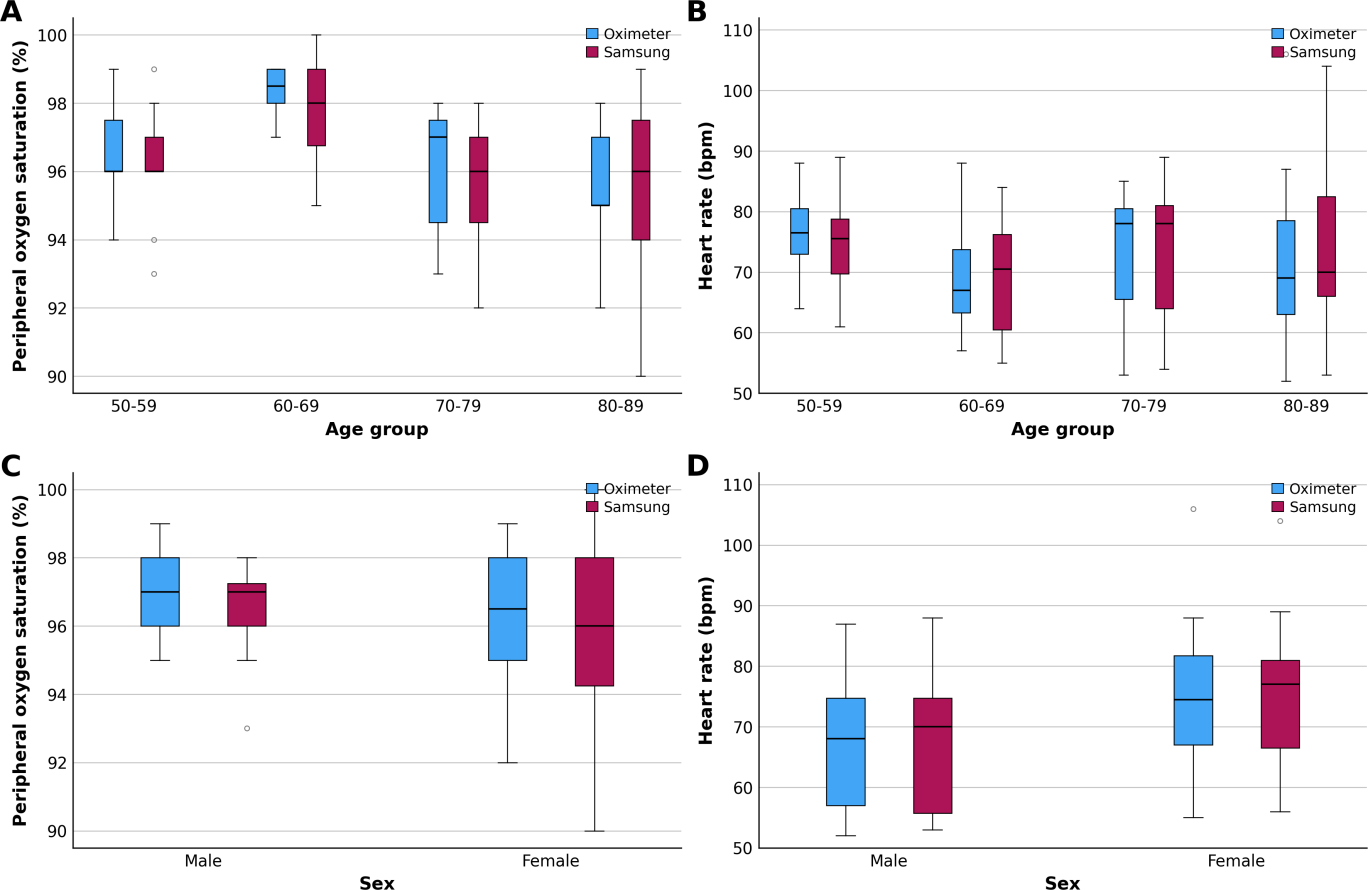
Boxplot of arterial oxygen saturation.

According to Figure 1, there appear to be no differences between age groups or sexes. As shown in Figure 3, the boxplots show similar distributions for the Samsung device and the reference pulse oximeter, with comparable medians and overlapping IQRs. Overall variability was similar between methods, as the first, second, and third quartiles, as well as minimum and maximum values, were largely consistent for both measurements.

**Figure 4.**
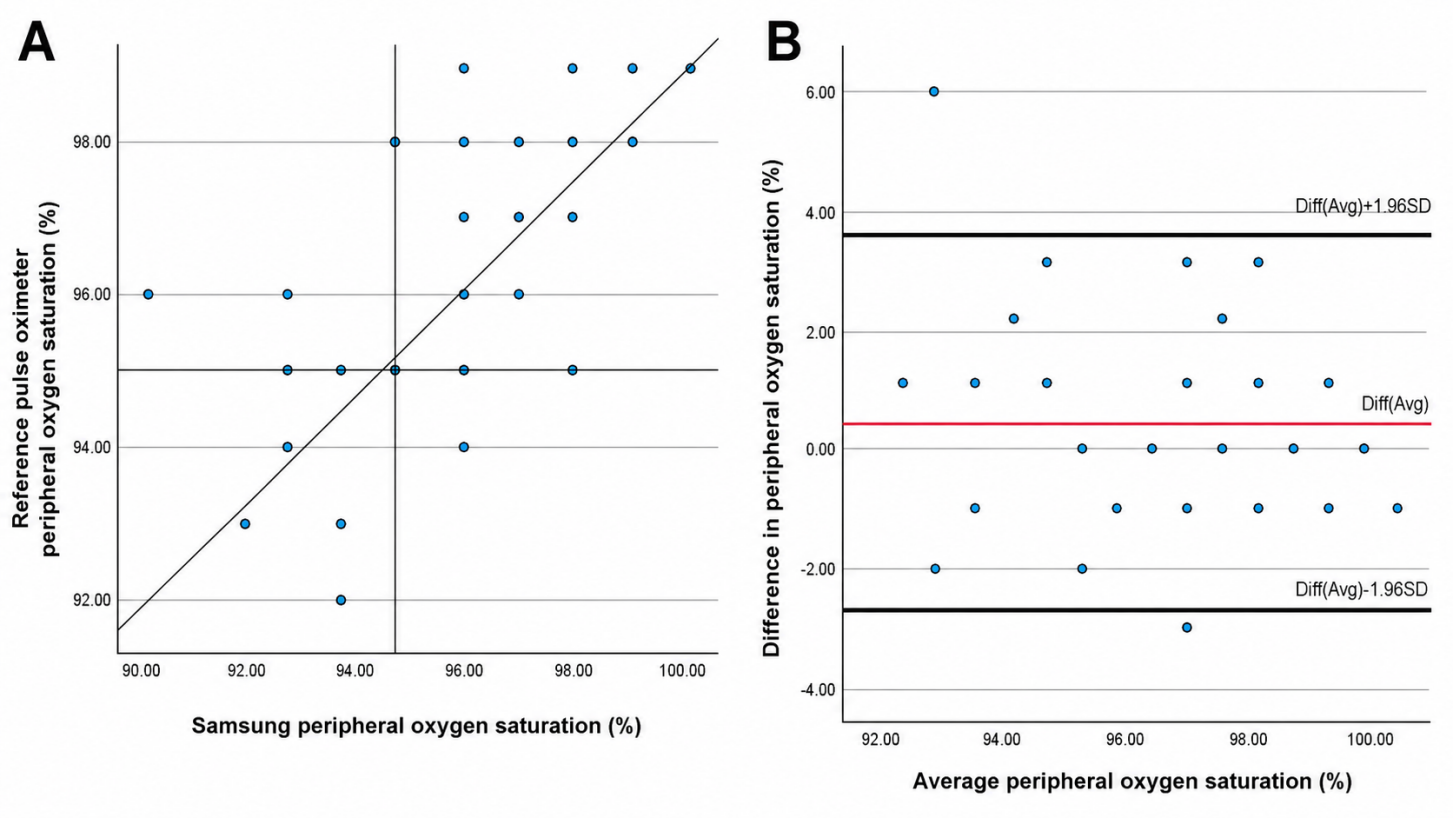
Scatterplots of arterial oxygen saturation. SpO_2_: peripheral oxygen saturation.

Visual inspection of the scatterplots (Figure 4) revealed a clear positive linear relationship between SpO₂ values measured by the Samsung device and those obtained with the reference pulse oximeter. Most data points were distributed close to the line of identity, indicating that the Samsung device generally follows the measurement trend of the reference method across the observed range of SpO₂ values. However, a moderate dispersion around the identity line was observed, which was more pronounced at lower SpO₂ levels, suggesting increased variability between the 2 methods in this range.

Bland-Altman analysis demonstrated a small positive mean bias, with the average difference remaining close to zero, indicating no clinically relevant systematic overestimation or underestimation by the Samsung device. The majority of measurements lay within the limits of agreement, supporting an overall acceptable level of agreement between the 2 methods. No clear evidence of proportional bias was identified, as the differences appeared relatively consistent across the range of mean SpO₂ values, and no increase in variability was observed at higher saturation levels.

Overall, these findings indicate good agreement between the Samsung device and the reference pulse oximeter at the group level. Nevertheless, the dispersion observed in both the scatterplot and the Bland-Altman plot suggests that individual SpO₂ measurements may differ between methods, particularly at lower saturation values, and this should be taken into account when interpreting single measurements. Taken together, Bland-Altman analyses and absolute difference distributions indicate acceptable agreement between devices at the group level under standardized resting conditions, while also highlighting interindividual variability that should be considered when interpreting individual measurements.

## Discussion

### Principal Findings

This study aimed to assess the reliability of the Samsung Galaxy Watch 6 in measuring BP and SpO₂ among individuals aged 50 to 89 years. Our findings suggest that the Samsung Galaxy Watch 6 demonstrates reliability in these measurements among individuals older than 50 years. The reliability of these data is crucial for facilitating the development of digital biomarkers and supporting clinical research endeavors involving monitoring [15]. Furthermore, this reliability is instrumental in the prevention and detection of adverse cardiac events [15,30]. Improved measurement reliability enhances the potential for wearables to serve as affordable and valuable tools for health monitoring, thereby promoting equitable access to health care [15,16,31,32].

It is important to emphasize that reliability in this context refers to agreement under standardized, calibrated, resting conditions, and should not be interpreted as full interchangeability with clinical-grade reference devices. Agreement between methods was, therefore, interpreted primarily based on Bland-Altman analysis, limits of agreement, and clinically meaningful absolute differences, rather than the absence of statistically significant mean differences.

Our findings indicate that the Samsung Galaxy Watch 6 reliably measures BP, showing a strong correlation compared to the reference method, with results falling within the range of good to excellent. This underscores the reliability of such technology for BP measurement among individuals aged 50 to 89 years. A previous study on the Samsung Galaxy Watch 4, a predecessor to the model used in our research, also demonstrated reliability in individuals aged 18 years, displaying a level of accuracy compared to the reference method [33]. Similarly, research on another wearable device, the Aktiia Bracelet, conducted on a population aged 2 to 65 years, revealed its safety and effectiveness in measuring BP compared to ambulatory blood pressure monitoring (ABPM) [34]. Recent reviews emphasize that smartwatch-based BP technologies should therefore be interpreted as complementary tools rather than substitutes for conventional clinical measurements [19].

In line with these recommendations, the findings indicate acceptable agreement at the group level, while Bland-Altman analysis revealed interindividual variability that should be considered when interpreting single measurements, particularly at higher BP values.

However, in another study validating BP measurement using a Samsung Galaxy Watch Active 2 smartwatch, a previous model used in our research, compared to ABPM, a systematic bias toward a calibration point was observed, resulting in an overestimation of low BP and an underestimation of high BP [19]. One contributing factor to these discrepancies is that the Samsung Galaxy Watch Active 2 smartwatch does not perform automatic measurements; instead, patients had to manually activate the smartwatch measurement for 24 hours following the ABPM measurement [19]. Additionally, differences in firmware versions among the same device model can lead to varying conclusions [9]. In this study, the assessments were meticulously controlled and conducted simultaneously using the Samsung Galaxy Watch 6 (SM-R930) and the TONOPORT V device.

The results of the measurement of SpO_2_ by the Samsung Galaxy Watch 6 smartwatch and the portable finger pulse oximeter appear to show a good level of agreement and positive correlation. Our results reinforce the reliability of smartwatches and the high accuracy of these devices compared to a standard pulse oximeter as evaluated in other studies [35,36]. In addition to the reliability of these wearables for use in clinical decision-making [35], smartwatches enable continuous and long-term monitoring [37]. These wearables can detect abnormal fluctuations and more quickly assess changes in the patient’s state of health over time [37]. In addition, this type of technology is particularly advantageous for some pathologies, such as chronic lung disease, sleep apnea, or post-COVID syndrome [37]. On the other hand, wearables take the burden off measurements taken in a medical environment and can minimize errors associated with anxiety induction and increased adrenergic activity in clinical settings, known as “hypertension or white coat syndrome” [4,9]. Moderate reliability indicates that, although the smartwatch may provide approximate SpO₂ estimates under controlled, normoxic resting conditions, its measurements should not be considered interchangeable with those obtained from clinical-grade pulse oximeters. Under these conditions, smartwatch-based SpO₂ measurements may be suitable for general trend monitoring in stable individuals but remain inadequate for clinical decision-making. It is important to note that part of the observed variability may be attributed to differences in the measurement site, as wrist-based assessments are more susceptible to variations in skin temperature and peripheral perfusion compared with finger-based pulse oximetry. Recent advances in wearable photoplethysmography sensor technology and signal processing have improved measurement performance; however, physiological factors, such as peripheral perfusion, skin temperature, and anatomical measurement site remain important sources of variability [15]. Contemporary reviews highlight that wrist-based photoplethysmography sensors are inherently more susceptible to these influences than finger-based pulse oximetry, reinforcing the cautious interpretation of smartwatch SpO₂ measurements [15].

When we validate portable devices, we have to be careful and follow a series of guidelines. In our study, we followed guidelines that can influence reproducibility and scientific rigor [9]. Bearing these guidelines in mind, our research attempted to verify the reliability of Samsung devices in different contexts, including sex and various age groups. Individual differences, sex variability, and demographics must be taken into account when collecting data using an optical sensor (photoplethysmography) [9]. Sex must be assessed to verify possible measurement errors between male participants and female participants [9], as some authors have reported a higher error rate in males than in females [37]. We did not find any significant differences between sexes with this smartwatch, possibly due to its more reliable function.

Much of the research on this topic does not take into account factors such as race and ethnicity, which can impair the observation and interpretation of health results [9]. Another guideline evaluated was skin tone, which should be taken into account when evaluating studies using wearables [9]. Due to the homogeneity of the population studied in terms of race, we did not encounter any difficulties in this regard. Another factor that can influence measurements is whether the participant has tattoos [9], which was not the case in our study. There also seems to be no consensus on the accuracy and absorption of green light in people with lighter skin tones [9]. Wearables that use green LED light have a shorter wavelength, so the amount of light that passes through the tissue can be limited [9]. Our research was carried out on a light-skinned Portuguese population without any tattoos in the area of the optical sensor. According to the results of our research, there were no significant changes in any of the variables evaluated. These results are in line with other studies that have found no differences in the use of optical green light sensors in people with lighter skin tones [31,38].

### Economic and Political Considerations of Health Care

The adoption of wearable devices has the potential to improve health care system efficiency by enabling the early detection of health problems, monitoring treatment outcomes, and reducing the burden on health care infrastructures [39-41]. Continuous monitoring through digital technologies may support more personalized and effective health management, potentially lowering costs related to hospitalizations and emergency care while improving health outcomes [39-41].

Wearable devices and mobile health platforms may also help reduce inequalities in access to health care by providing affordable monitoring solutions, particularly in resource-limited settings, thereby promoting more equitable health care delivery [41]. Overall, these technologies have the potential to enhance both individual and population health while optimizing health care resources.

### Study Limitations and Future Research

This study has several limitations that should be acknowledged, including the relatively small sample size, the homogeneous demographic characteristics of the study population, and the fact that all measurements were conducted on the same day and under resting conditions only, which may not reflect BP variability during daily activities. The study included only 50 participants from a Caucasian Portuguese population, which limits the generalizability of the findings to other ethnicities or populations. Therefore, the results should be interpreted with caution and should not be extrapolated beyond similar demographic contexts without further validation.

An additional limitation is that smartwatch calibration and validation were performed using the same reference BP device within a single session, which may partially inflate agreement and does not allow for the assessment of calibration stability across days. Another limitation is that BP was measured on contralateral arms, and interarm differences may have contributed to measurement variability; therefore, findings should be interpreted as an arm-to-arm comparison under standardized resting conditions.

Therefore, future studies are recommended to (1) assess the reliability of the wearable for BP measurement at different times of the day; (2) evaluate BP measurement reliability after more intense daily activities; (3) examine reliability across different days; (4) verify smartwatch-based SpO₂ measurements using repeated assessments; (5) assess SpO₂ reliability during movement and daily activities; (6) evaluate SpO₂ measurements across a broader range of oxygen saturation values, including hypoxemic conditions; and (7) investigate the influence of BMI and other individual factors, such as skin tone, on measurement accuracy. Future validation protocols should also incorporate calibration and validation sessions performed on different days and assess device performance during unsupervised, real-world use.

Wearable devices may represent a cost-effective and practical approach for collecting longitudinal BP data and constructing individual BP profiles. However, to fully realize this potential, wearable technologies should be capable of continuous and autonomous BP monitoring. The smartwatch used in this study does not currently support fully automatic BP measurements, which represents an additional limitation.

### Conclusions

This study assessed the agreement between the Samsung Galaxy Watch 6 and reference devices for BP and SpO₂ measurements in adults aged 50 years and older. The findings indicate that the smartwatch demonstrates good agreement with the reference method for SBP and DBP when averaged across repeated measurements, despite statistically significant differences observed in some individual measurement sessions. These results suggest that the device may be suitable for resting BP monitoring based on repeated measurements, consistent with standard clinical practice.

For SpO₂, the smartwatch showed moderate agreement with the reference pulse oximeter, with no significant systematic bias. This level of agreement supports its use for nonclinical monitoring and trend observation under resting conditions.

In summary, the Samsung Galaxy Watch 6 demonstrated acceptable agreement with reference devices for the assessment of BP and SpO₂ in older adults without decompensated clinical conditions, evaluated under controlled resting conditions. These findings indicate that the device provides reliable measurements within this specific population and context, thereby supporting its use as a complementary assessment tool when measurements are obtained under standardized and physiologically stable conditions.

## Abbreviations

ABPM: ambulatory blood pressure monitoring
BP: blood pressure
DBP: diastolic blood pressure
ES: effect size
HR: heart rate
ICC: intraclass correlation coefficient
SBP: systolic blood pressure
SpO_2_: peripheral oxygen saturation
WHO: World Health Organization

## References

[1] Katz ME, Mszar R, Grimshaw AA (2024). Digital health interventions for hypertension management in US populations experiencing health disparities: a systematic review and meta-analysis. JAMA Netw Open.

[2] Zhang D, Lee JS, Pollack LM (2024). Association of economic policies with hypertension management and control: a systematic review. JAMA Health Forum.

[3] John O, Campbell NRC, Brady TM (2021). The 2020 “WHO technical specifications for automated non-invasive blood pressure measuring devices with cuff”. Hypertension.

[4] Mills KT, Stefanescu A, He J (2020). The global epidemiology of hypertension. Nat Rev Nephrol.

[5] Sierra C (2017). La hipertensión arterial en el anciano [Article in Spanish]. Hipertens Riesgo Vasc.

[6] Stavropoulos TG, Papastergiou A, Mpaltadoros L, Nikolopoulos S, Kompatsiaris I (2020). IoT wearable sensors and devices in elderly care: a literature review. Sensors (Basel).

[7] Janson P, Willeke K, Zaibert L (2022). Mortality, morbidity and health-related outcomes in informal caregivers compared to non-caregivers: a systematic review. Int J Environ Res Public Health.

[8] Bherer L (2015). Cognitive plasticity in older adults: effects of cognitive training and physical exercise. Ann N Y Acad Sci.

[9] Nelson BW, Low CA, Jacobson N, Areán P, Torous J, Allen NB (2020). Guidelines for wrist-worn consumer wearable assessment of heart rate in biobehavioral research. NPJ Digit Med.

[10] Singhal A, Prafull K, Daulatabad VS, John NA, John J (2023). Arterial oxygen saturation: a vital sign?. Niger J Clin Pract.

[11] Ahmad NA, Mat Ludin AF, Shahar S, Mohd Noah SA, Mohd Tohit N (2020). Willingness, perceived barriers and motivators in adopting mobile applications for health-related interventions among older adults: a scoping review protocol. BMJ Open.

[12] Flandorfer P (2012). Population ageing and socially assistive robots for elderly persons: the importance of sociodemographic factors for user acceptance. Int J Popul Res.

[13] Dogra S, Dunstan DW, Sugiyama T, Stathi A, Gardiner PA, Owen N (2022). Active aging and public health: evidence, implications, and opportunities. Annu Rev Public Health.

[14] Castaneda D, Esparza A, Ghamari M, Soltanpur C, Nazeran H (2018). A review on wearable photoplethysmography sensors and their potential future applications in health care. Int J Biosens Bioelectron.

[15] Dcosta JV, Ochoa D, Sanaur S (2023). Recent progress in flexible and wearable all organic photoplethysmography sensors for SpO2 monitoring. Adv Sci (Weinh).

[16] Munos B, Baker PC, Bot BM (2016). Mobile health: the power of wearables, sensors, and apps to transform clinical trials. Ann N Y Acad Sci.

[17] Nelson BW, Allen NB (2019). Accuracy of consumer wearable heart rate measurement during an ecologically valid 24-hour period: intraindividual validation study. JMIR mHealth uHealth.

[18] Ienca M, Jotterand F, Elger B (2017). Intelligent assistive technology for Alzheimer’s disease and other dementias: a systematic review. J Alzheimers Dis.

[19] Falter M, Scherrenberg M, Driesen K (2022). Smartwatch-based blood pressure measurement demonstrates insufficient accuracy. Front Cardiovasc Med.

[20] Siu AL, U.S. Preventive Services Task Force (2015). Screening for high blood pressure in adults: U.S. Preventive Services Task Force recommendation statement. Ann Intern Med.

[21] Leung AA, Nerenberg K, Daskalopoulou SS (2016). Hypertension Canada’s 2016 Canadian Hypertension Education Program guidelines for blood pressure measurement, diagnosis, assessment of risk, prevention, and treatment of hypertension. Can J Cardiol.

[22] O’Brien E, Parati G, Stergiou G (2013). European Society of Hypertension position paper on ambulatory blood pressure monitoring. J Hypertens.

[23] Jones NR, McCormack T, Constanti M, McManus RJ (2020). Diagnosis and management of hypertension in adults: NICE guideline update 2019. Br J Gen Pract.

[24] Shimamoto K, Ando K, Fujita T (2014). The Japanese Society of Hypertension guidelines for the management of hypertension (JSH 2014). Hypertens Res.

[25] Liu LS, Writing Group of 2010 Chinese Guidelines for the Management of Hypertension (2011). 2010 Chinese guidelines for the management of hypertension. Zhonghua Xin Xue Guan Bing Za Zhi [Article in Chinese].

[26] O’Brien E, White WB, Parati G, Dolan E (2018). Ambulatory blood pressure monitoring in the 21st century. J Clin Hypertens (Greenwich).

[27] Haensel A, Utech K, Langewitz W (2005). Validation of TONOPORT V blood-pressure measuring monitor in adults. J Hum Hypertens.

[28] (2023). GE TONOPORT V operator´s manual. https://medaval.ie/docs/manuals/GE-Tonoport-V-Manual.pdf.

[29] Chan ED, Chan MM, Chan MM (2013). Pulse oximetry: understanding its basic principles facilitates appreciation of its limitations. Respir Med.

[30] Prawiro E, Yeh CI, Chou NK, Lee MW, Lin YH (2016). Integrated wearable system for monitoring heart rate and step during physical activity. Mob Inf Syst.

[31] Bent B, Goldstein BA, Kibbe WA, Dunn JP (2020). Investigating sources of inaccuracy in wearable optical heart rate sensors. NPJ Digit Med.

[32] Weiss D, Rydland HT, Øversveen E, Jensen MR, Solhaug S, Krokstad S (2018). Innovative technologies and social inequalities in health: a scoping review of the literature. PLoS One.

[33] Lins LFTS, do Nascimento EGC, da Silva Júnior JA, de Medeiros Fernandes TAA, de Andrade MF, de Mesquita Andrade C (2023). Accuracy of wearable electronic device compared to manual and automatic methods of blood pressure determination. Med Biol Eng Comput.

[34] Vybornova A, Polychronopoulou E, Wurzner-Ghajarzadeh A, Fallet S, Sola J, Wuerzner G (2021). Blood pressure from the optical Aktiia Bracelet: a 1-month validation study using an extended ISO81060-2 protocol adapted for a cuffless wrist device. Blood Press Monit.

[35] Khattak AF, Kakakhel SS, Wazir NK, Khattak M, Khattak T, Akbar F (2021). Reliability of smartphone applications for the quantification of oxygen saturation. Cureus.

[36] Walzel S, Mikus R, Rafl-Huttova V, Rozanek M, Bachman TE, Rafl J (2023). Evaluation of leading smartwatches for the detection of hypoxemia: comparison to reference oximeter. Sensors (Basel).

[37] Shcherbina A, Mattsson CM, Waggott D (2017). Accuracy in wrist-worn, sensor-based measurements of heart rate and energy expenditure in a diverse cohort. J Pers Med.

[38] Wallen MP, Gomersall SR, Keating SE, Wisløff U, Coombes JS (2016). Accuracy of heart rate watches: implications for weight management. PLoS One.

[39] Kario K (2020). Management of hypertension in the digital era: small wearable monitoring devices for remote blood pressure monitoring. Hypertension.

[40] Howard M (2021). Wearables, the marketplace and efficiency in healthcare: how will I know that you’re thinking of me?. Philos Technol.

[41] De Sario Velasquez GD, Borna S, Maniaci MJ (2024). Economic perspective of the use of wearables in health care: a systematic review. Mayo Clin Proc Digit Health.

